# 732 Examining Staff Views and Perceptions After Implementation of a Laser Doppler Imaging Device Into Practice

**DOI:** 10.1093/jbcr/irac012.286

**Published:** 2022-03-23

**Authors:** Shawn Tejiram, Thomas Washburn, Jeffrey W Shupp

**Affiliations:** MedStar Washington Hospital Center, Kensington, Maryland; MedStar Washington Hospital Center, Washington, District of Columbia; MedStar Washington Hospital Center, Washington DC, District of Columbia

## Abstract

**Introduction:**

Laser doppler imaging (LDI) has been established as an accurate diagnostic tool to assess burn depth and measure healing potential. Despite this, its use in burn centers remain limited. While studies have examined challenges to LDI use, there is a paucity of literature factoring staff views and attitudes as a barrier to implementation into burn practice. The aim of this work was to assess and examine attitudes and perspectives following implementation of an LDI protocol into acute burn injury assessment.

**Methods:**

Following institutional approval, a 22-question survey was disseminated among staff involved with implementation of an LDI device as a point of care tool to assess acute burn injury at a single ABA verified burn center. The survey focused on questions examining device ease of use, understanding of the underlying LDI technology, interpretation of imaging generation from wounds, and perceptions of patients’ experience. Questions were answered on a standard 5-point Likert scale. All survey data was collected anonymously into an electronic database for assessment.

**Results:**

Overall, there were 15 respondents to the survey questions. Five respondents found the LDI device difficult to use (33%). Barriers to device ease of use included difficulty with device movement (60%), incorporation of scanning into wound care and dressing placement (60%), and management of hardware or software issues that arise during use (60%). Challenges noted by respondents external to device use was mainly high patient census (80%). Despite this, 60% of respondents found the device easier to use after performing several scans in one or more patients and 60% found that scans generated matched their assessment of burn depth. Among respondents, 66% found patients amenable to the scanning process and 80% did not feel that that the scanning process worsened patients’ pain.

**Conclusions:**

Challenges in LDI device use, implementation with wound care and dressing placement, and high patient census were identified as barriers to LDI use. Despite this, ease of device use improved with more frequent use. Identification of views and perceptions such as these can lead to protocol changes and additional training that facilitate ease of LDI use. Further examination will be required to better elucidate this information.

